# The Diagnosis Value of Promoter Methylation of UCHL1 in the Serum for Progression of Gastric Cancer

**DOI:** 10.1155/2015/741030

**Published:** 2015-10-15

**Authors:** Gongping Wang, Wei Zhang, Bo Zhou, Canhui Jin, Zengfang Wang, Yantong Yang, Zhenzhen Wang, Ye Chen, Xiaoshan Feng

**Affiliations:** Department of Gastrointestinal Oncology Surgery, The First Affiliated Hospital, Henan University of Science and Technology, Luoyang, Henan 471003, China

## Abstract

*Background.* Aberrant promoter methylation has been considered as a potential molecular marker for gastric cancer (GC). However, the role of methylation of FLNC, THBS1, and UCHL1 in the development and progression of GC has not been explored. *Methods.* The promoter methylation status of UCHL1, FLNC, THBS1, and DLEC1 was assessed by quantitative methylation-specific PCR (QMSP) in the serum of 82 GC patients, 46 chronic atrophic gastritis (CAG) subjects, and 40 healthy controls. *Results.* All four genes had significantly higher methylation levels in GC patients than in CAG and control subjects. However, only UCHL1 methylation was significantly correlated with the tumor stage and lymph node metastasis. While THBS1 methylation was altered in an age-dependent manner, FLNC methylation was correlated with differentiation and *Helicobacter pylori* infection. DLEC1 methylation was only associated with tumor size. Moreover, methylated UCHL1 with or without THBS1 in the serum was found to be significantly associated with a poor prognosis. *Conclusion.* The promoter methylation degree of FLNC, THBS1, UCHL1, and DLEC1 in serum could tell the existence of GC and only UCHL1 in the serum was also associated with poor prognosis of GC.

## 1. Introduction

Gastric cancer is one of the most frequently diagnosed malignancies worldwide, particularly in China [1]. Despite improvements in therapy over the past few decades, the low detection rate in the early stage of GC is the major cause of the high mortality rate in the GC patients [2]. Evidence accumulated for decades has revealed that GC is an end result of the multistep transformation of precancerous gastric lesions, including CAG [3]. Moreover,* H. pylori* infection plays a critical role in GAC and GC pathogenesis [4]. Thus, exploring the epigenetic abnormities involved in the process of GC would contribute to the understanding of the course of GC tumorigenesis, and searching for specific markers for early diagnosis and prognosis estimation may benefit the survival rates of patients.

DNA methylation refers to the addition of a methyl group to the carbon 5′ position of the cytosine ring of CpG dinucleotides to form 5-methylcytosine. CpG dinucleotides are concentrated in the upstream promoter region of many genes [5]. The promoter methylation of genes involved in DNA repair, cell-cycle control, apoptosis, and cell adhesion has already been confirmed in various tumor types, including GC [4–6]. Using methylated genes as a molecular marker may provide powerful diagnostic and prognostic value in gastric cancer patients.

It is now widely accepted that the majority of the circulating DNA in cancer patients originated from neoplastic cells and resulted from DNA fracture due to apoptosis or necrosis of tumor cell. Therefore, the circulating DNA has a similar methylation pattern as that in the primary tumor, indicating that circulating methylated DNA in the serum has the potential as a novel diagnostic biomarker for cancer. Different study groups have described the promoter methylation frequency of DAPK, E-cadherin, GSTP1, p16, TIMP3, and APC in the serum of GC patients [7, 8]. And four genes including FLNC, THBS1, UCHL1, and DLEC1 were reported to be hypermethylated in tissue specimens of GC patients [9–12].

Therefore, in this study, we investigated the promoter methylation in the serum DNA for FLNC, THBS1, UCHL1, and DLEC1 in 82 GC patients, 46 CAG subjects, and 40 healthy controls with clinicopathological factors to assess their value in diagnosis or prognosis for GC patients. We indicated that the promoter methylation level of all the four genes in the serum of GC patients was higher and of them UCHL1 was valuable for diagnosis of GC progression while it was correlated with poor prognosis.

## 2. Materials and Methods

### 2.1. Ethics Statement

Institutional Ethics Board approval was obtained from the Medical Ethics Committee of the Henan University of Science and Technology. All participating patients were formally informed for the purpose of using their medical records and the written informed consents were obtained from all participants in this clinical trial.

### 2.2. Patients and Samples

Serum samples from 82 GC and 46 CAG patients were obtained between Jan. 2012 and Jan. 2013 at Department of Gastrointestinal Oncology Surgery, The First Affiliated Hospital, Henan University of Science and Technology. None of the enrolled GC patients had received preoperative chemotherapy, immunotherapy, or radiation therapy. 46 patients with CAG were diagnosed by gastroscopic examination, and 40 age-matched healthy subjects without a previous history of cancer were simultaneously recruited from the individuals who visited the hospital. All blood samples were acquired before any therapeutic intervention. The TNM stage was assessed according to the criteria of the Union for International Cancer Control [17]. Tumor differentiation and histological type were confirmed by pathological examination. Alcohol consumption was defined as the intake of more than 2 alcoholic drinks per day continuously for at least half a year. GC patients were regularly followed up in our department or were interviewed by telephone. The mean age was 63.3 ± 7.01 years (45–78 years) for the GC patients, 61.4 ± 4.14 years (50–69 years) for the CAG patients, and 58.4 ± 4.11 years (48–66 years) for the healthy controls. The* H. pylori* infection status in GC patients was tested by serologic and histological analyses or by the urea breath test. All blood samples were centrifuged at 2000 ×g for 10 minutes and stored at −80°C until the DNA was extracted.

### 2.3. DNA Extraction and Bisulfite Treatment

Genomic DNA was extracted from a total of 1000 *μ*L serum by using a QIAamp Blood Mini Kit (Qiagen, Hilden, Germany) according to the manufacturer's directions. Then, the DNA was modified by sodium bisulfite to convert unmethylated cytosines to uracils with an EpiTect Bisulfite Kit (Qiagen).

### 2.4. Quantitative Methylation-Specific PCR

The bisulfite-modified DNA was used as a template for fluorescence-based real-time PCR, as previously described [18]. In brief, 10 ng of bisulfite-modified DNA was used as the template in QMSP assays, which were carried out in a final volume of 20 *μ*L in 384-well plates in the LightCycler 480 (Roche, Basel, Switzerland). PCR was performed in separate wells for each primer set, and each sample was run in triplicate. The final reaction mixture contained 600 nmol/L of each primer (Invitrogen, Carlsbad, CA), 1 unit of platinum Taq polymerase (Invitrogen), a 200 *μ*mol/L concentration each of dATP, dCTP, dGTP, and dTTP, 16.6 mmol/L ammonium sulfate, 67 mmol/L Trizma, 6.7 mmol/L magnesium chloride, 10 mmol/L mercaptoethanol, and 1 × SYBR Green I dye (Sigma-Aldrich, St. Louis, MO, USA). PCR was conducted under the following conditions: 1 cycle at 95°C for 5 min followed by 45 cycles at 95°C for 10 sec, 58°C for 10 sec, 72°C for 20 sec, and 81°C for 1 sec. Leukocyte DNA from a healthy individual was methylated in vitro with excess SssI methyltransferase (New England Biolabs, Beverly, MA) to generate completely methylated DNA as a positive control. Each plate included subject DNA samples, water blanks, and serial dilutions (30–0.003 ng) of the positive control, which were used to construct a calibration curve.

The relative methylation level of the 4 genes in each sample was calculated as the ratio of the amplified gene of interest to ACTB and was then multiplied by 1000 for easier tabulation (the average value of triplicates of each gene of interest divided by the average value of triplicates of ACTB  ×  1000). The primers, obtained from the literature, specifically amplified the promoter regions of the 4 genes of interest and the internal control gene ACTB (Table 1).

### 2.5. Statistical Analysis

Statistical analysis was performed using the SPSS 16.0 (IBM, Armonk, NY) and Prism 5 (GraphPad Software, San Diego, CA) software. The Kruskal-Wallis test was used to compare differences in methylation levels among the three groups (control, CAG, and GC), and the Bonferroni correction was used for paired comparisons. The methylation thresholds of each gene for the cut-off values were determined by receiver operating characteristic (ROC) analysis. The cut-off value was used to establish the gene methylation status (positive or negative). The correlations between the clinicopathological characteristics of GC patients and the gene methylation status in the serum were examined by Fisher's exact test. Overall survival analysis was performed by using the Kaplan-Meier method, and the log-rank test was used to compare the differences in the survival curves. Those patients who were lost to follow up were excluded from the survival analysis. Cox's proportional hazard regression analysis was used to analyze the hazard ratio (HR) and 95% confidence interval (CI) of independent factors for patients' survival. A *p* value of less than 0.05 was considered to be statistically significant.

## 3. Results

### 3.1. Methylation Levels in the Serum of Subjects

**Figure 1:**
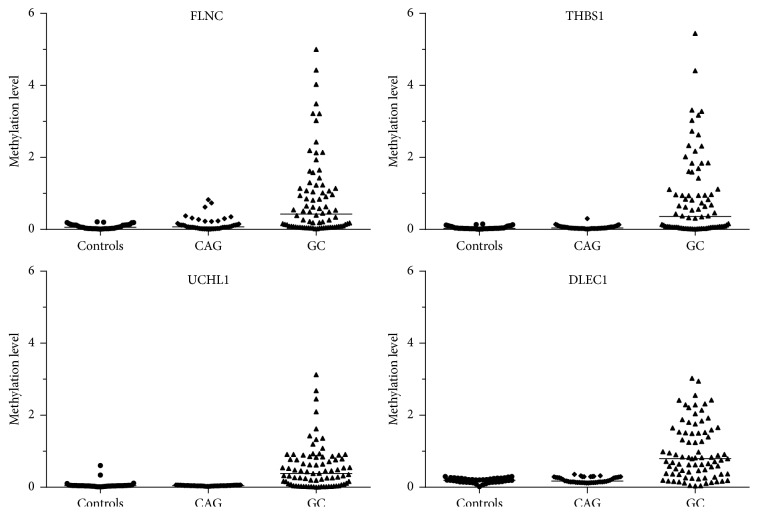
Promoter methylation levels for the 4 markers in serum DNA from gastric cancer (GC), chronic atrophic gastritis (CAG), and healthy controls.

The serum DNA methylation levels of four genes in GC subjects were significantly higher than those of the control and CAG subjects (Figure 1). Overall significant differences in the methylation levels were observed for UCHL1 (*p* < 0.001), FLNC (*p* = 0.005), THBS1 (*p* < 0.001), and DLEC1 (*p* = 0.012) (Table 2). No significant differences were found between the controls and CAG subjects for any of the gene methylation levels. All four genes showed significantly higher methylation levels in GC patients than in controls (*p* = 0.002 for FLNC, *p* < 0.001 for THBS1, *p* < 0.001 for UCHL1, and *p* = 0.005 for DLEC1).

### 3.2. Methylation Frequency in the Serum of Subjects

Considering the similarity of the methylation levels between healthy controls and CAG patients, these subjects were merged for the next step of the analyses. We calculated the specificities, sensitivities, and areas under the curves (AUCs) of the four genes (Table 3).

**Figure 2:**
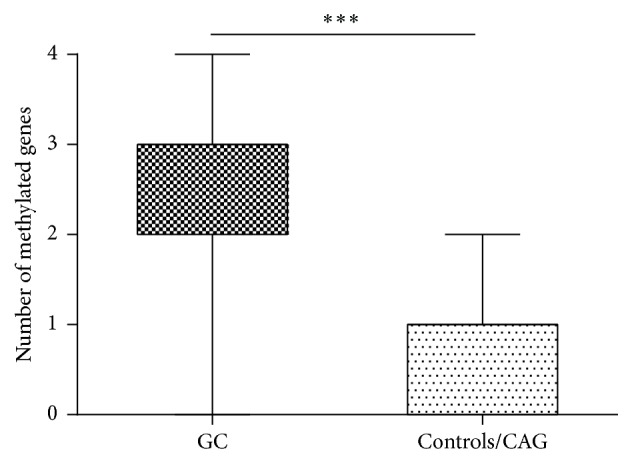
Average methylated-genes numbers are significantly different between GC (2.68 ± 0.92, 95% CI = 2.47–2.87) and CAG/controls (0.68 ± 0.69, 95% CI = 0.54–0.83) (^*∗∗∗*^ *p* < 0.001).

The frequency of detecting the methylated gene in the serum of GC patients was 67% (55/82) for FNLC, 63.4% (52/82) for THBS1, 56.1% (46/82) for UCHL1, and 80.5% (66/82) for DLEC1. Of the 82 GC patients with results for the four genes, 41.5% (34/82) showed two methylated genes in the serum and 30.5% (25/82) showed three methylated genes in the serum. In contrast, 45% (18/40) of controls and 43.5% (20/46) of CAG subjects showed no methylation of any gene and 42.5% (17/40) of controls and 43.5% (20/46) of CAG subjects harbored only one methylated gene. The mean value of methylated genes in the serum of GC patients was significantly increased (2.68 ± 0.92, 95% CI = 2.47–2.87) compared with that of the controls/CAG samples (0.68 ± 0.69, 95% CI = 0.54–0.83) (*p* < 0.001, Figure 2). In a word, the specificity of each gene was about 90%, while the sensitivity was within a range from 56.1% to 80.5%.

The above data demonstrated that the promoters of the four genes were more frequently overmethylated in GC patients but not in controls or CAG samples. So the overmethylated promoter in the four genes could tell the existence of GC.

### 3.3. Correlation of the Serum Methylation Status with Clinicopathological Data

The methylation status of UCHL1, THBS1, FLNC, and DLEC1 was analyzed for correlations with clinicopathological characteristics of GC patients. Our data showed that UCHL1 methylation was significantly correlated with the TNM stage (*p* = 0.026) and lymph node metastasis (*p* = 0.014). THBS1 methylation was correlated with age greater than 60 years (*p* = 0.021), whereas FLNC methylation was associated with differentiation (*p* = 0.016) and* H. pylori* infection (*p* = 0.009). DLEC1 methylation was positively associated with tumor size only (*p* = 0.04). There were no other significant associations between gene methylation and the clinicopathological features of GC patients. All results are shown in Table 4.

### 3.4. Methylation Status of Genes and Overall Survival

**Figure 3:**
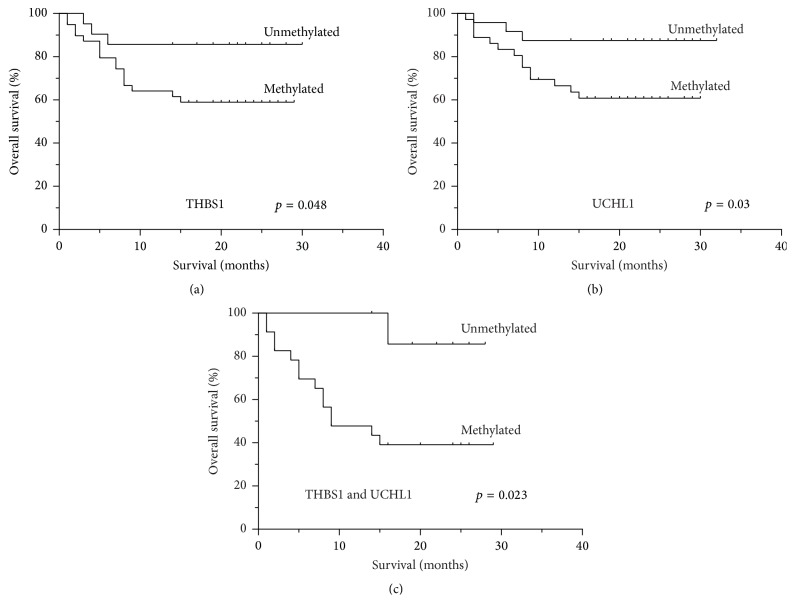
Kaplan-Meier analysis of the percentage of overall survival in GC patients according to methylation status. (a) THBS1 methylation (*p* = 0.048). (b) UCHL1 methylation (*p* = 0.03). (c) Combined THBS1 and UCHL1 (*p* = 0.023).

The associations between clinical outcomes and the serum methylation status of FLNC, THBS1, UCHL1, and DLEC1 were analyzed in GC patients for whom complete follow-up information was available. GC patients without methylated UCHL1 tended to have a better survival (median survival of 24 months) than those with methylated UCHL1 (median survival of 15 months) (*p* = 0.03, Figure 3(a)), while the overall median survival of GC patients with or without methylated THBS1 in the serum was 14 or 20 months, respectively (*p* = 0.048, Figure 3(b)). However, the presence of methylated FLNC and DLEC1 in the serum was not associated with the patients' overall survival (*p* = 0.21 and *p* = 0.38, resp.).

What is more, GC patients with neither methylated THBS1 nor methylated UCHL1 in the serum showed a significantly better prognosis compared with those with both methylated THBS1 and methylated UCHL1 (median survival 9 versus 20 months, resp., *p* = 0.023, Figure 3(c)). In contrast, other combinations of methylated genes were not found to be correlated with the patients' overall survival.

### 3.5. Analysis of Prognostic Significance of Clinical Variables

The association of clinicopathological data and serum methylation status of the genes FLNC, THBS1, UCHL1, and DLEC1 with clinical outcome was analyzed in GC patients. Statistical analysis revealed prognostic significance for the age above 60 years, poor differential, and higher TNM stage (*p* = 0.034, 0.011, and 0.000, resp., Table 5). The prognosis of patients with methylation of UCHL1 in serum was associated with a relative risk of death of 6.694 (95% CI, 1.536–19.172; *p* = 0.011).

The multivariate analysis included age, differential, TNM stage, and methylation of UCHL1. In the Cox proportional hazards regression analysis of independent variables age (≥60 years), differential (poor), and methylation of UCHL1 did not attain statistical significance (*p* = 0.990, 0.095, and 0.103, resp., Table 5). Only higher TNM stage was found to provide independent prognostic information correlated with a relative risk of death of 10.799 (95% CI, 2.013–37.938; *p* = 0.005).

## 4. Discussion

Gastric cancer is a major health problem worldwide. An increasing number of studies have shown that gastric cancer is a multistep process involving epigenetic alterations. Promoter methylation is a key epigenetic mechanism that participates in regulating gene expression and has been frequently observed in almost all types of human malignancies, including GC. The detection of methylated DNA in the serum provides a promising method for the noninvasive diagnosis of GC. However, the majority of data have been from the analysis of commonly methylated genes in the serum of patients with gastric cancer such as RUNX3 and p16 which were shown to be helpful for detection of gastric cancer [19, 20]. Therefore, we investigated novel serum methylation markers with potential diagnostic or prognostic value in GC patients.

In the present study, we detected the promoter methylation level of four genes, FLNC, THBS1, UCHL1, and DLEC1, in a cohort of GC patients using the QMSP method. All the four genes have been reported to be hypermethylated in the specimens of GC patients. FLNC, a member of the filamin family, has been known to organize actin polymerization in response to various signals [21]. Aberrant promoter methylation of FLNC in gastric cancer was initially found by the genome scanning technique [9]. Our data indicated that the frequency of FLNC methylation in the serum of GC patients was 67%. UCHL1, also called protein gene product 9.5 (PGP9.5), is a member of the ubiquitin carboxyl-terminal hydrolase family and plays a role in the development of many tumor types via aberrant promoter methylation [22, 23]. Similarly to the reports of other groups, for example, that 64.9% of serum samples had methylated UCHL1 methylation in nasopharyngeal carcinoma [24], the methylation frequency of UCHL1 in the present study was 56.1%. In addition, a significant difference in the serum DNA methylation level of UCHL1 between GC patients and healthy controls was found, in accordance with previous studies of gastric tissue specimens [10, 25]. DLEC1 is a common tumor suppressor gene that is often downregulated or lost in lung cancer due to promoter hypermethylation [26]. Compared with a former study documenting that DLEC1 methylation was detected in 33.8% of gastric adenocarcinoma serum samples by methylation-specific PCR [12], our result (80.5%) was much higher. The difference in the sensitivity of the detection method may be a possible explanation for this discrepancy. In other tumor types, DLEC1 was found to be methylated in the serum of 25% of nasopharyngeal carcinoma patients and in the plasma of 35.9% of NSCLC patients [24, 26]. Differences in the tumor type may explain the variety in the serum methylation frequency of DLEC1. THBS1 is a potential angiogenesis inhibitor that is frequently methylated in colorectal cancer and malignant glioma [27–29]. In the serum of GC patients, we observed that 63.4% of samples had methylated THBS1, which was slightly higher than previous studies reporting that the methylation frequency in GC and colorectal cancer tissues samples was 48.4% and 44.4%, respectively [11, 28]. Overall, the genes involved in this study exhibited a significantly higher methylation level or frequency in the serum samples of GC patients, which, for the most part, corresponded to previous data from other groups.

Considering that gene methylation plays an essential role in tumorigenesis, we further explored the clinicopathological significance and prognostic value in GC patients. Compared with early stage GC or GC without lymph node metastasis, UCHL1 was more frequently methylated in advanced stage GC. Our results confirm the results of previous studies in GC [30], colon cancer [31], and esophageal squamous cell carcinoma [32]. These results suggested that UCHL1 methylation may contribute to tumor progression in late stage GC patients. Furthermore, UCHL1 methylation was positively associated with worse overall survival rates, which is consistent with former studies in breast cancer [33], esophageal squamous cell carcinoma [32], and renal cell carcinoma [34]. Therefore, UCHL1 may be very valuable for prognosis of GC patients. In addition, the THBS1 methylation status was also associated with survival time. Similarly, other reports found a significant association between THBS1 methylation and the survival time of patients with penile squamous cell carcinoma and malignant glioma [29, 35]. GC patients over 60 years old showed more frequent THBS1 methylation than patients aged less than 60 years. Kang GH's study reported that the methylation frequency of THBS1 also increased with age in normal gastric mucosa [36]. These results suggest that THBS1 is methylated in an age-dependent manner. For the methylation status of DLEC1, we found a significantly higher level of DLEC1 methylation in large-sized tumors, which is consistent with a previous study [37]. Thus, DLEC1 methylation can be associated with tumor growth. No prognostic value was detected for DLEC1 methylation in our study or in a research report studying colorectal cancer [38]; however, a study in lung cancer provided the opposite view [39]. Whether the DLEC1 methylation status is correlated with the prognosis of cancer patients requires further retrospective studies. In addition, methylated FLNC was correlated with poorly differentiated GC. The underlying mechanism for the association of FLNC methylation and tumor cell differentiation requires further research. More importantly, FLNC methylation frequently occurred in GC patients with* H. pylori* infections. Of interest, several studies have reported that the promoter methylation level was significantly increased in the* H. pylori*-infected gastric mucosa of healthy people [40]. Thus, we suspected that FLNC methylation may be the result of* H. pylori* infection in GC patients.

In conclusion, using a QMSP approach, we identified a panel of methylated genes in the serum that differentiates GC patients from healthy controls and CAG patients and confirmed that the gene methylation status was markedly correlated with special clinicopathological features. Moreover, THBS1 and UCHL1 methylation in the serum was closely correlated with worse clinical outcomes in gastric cancer patients. Thus, FLNC, THBS1, UCHL1, and DLEC1 may be useful for detection of GC. And UCHL1 was a valuable diagnostic marker for progression of GC while it was also associated with the prognosis of gastric cancer patients.

## Figures and Tables

**Table 1:** Primers used for PCR amplification of interested genes.

Gene	Primer (5′-3′)	Annealing temperature (°C)	Reference
FLNC	F: GAGAGAGAGTTAGAGAGCGGTCGAGC R: GACCACGAAACTCGCTACGCTACG	64	[13]

DLEC1	F: GTTTCGTAGTTCGGTTTCGTC R: CGAAATATCTTAAATACGCAACG	58	[14]

THBS1	F: TGCGAGCGTTTTTTTAAAAGC R: TAAACTCGCAAACCAACTCG	60	[15]

UCHL1	F: GGTTCGGTCGTATTATTTCGC R: ACTACATCTTCGCGAAACGCCCG	62	[16]

**Table 2:** Methylation level of four genes in the serum of age-matched controls, CAG patients, and GC patients.

Gene	Methylation level (mean ± SD)			*p* value			
	Controls	CAG	GC	Overall^a^	Controls versus CAG^b^	Controls versus GC^b^	CAG versus GC^b^
	(*n* = 40)	(*n* = 46)	(*n* = 82)				
FLNC	0.082 ± 0.063	0.147 ± 0.184	0.849 ± 1.100	0.005	0.309	0.002	0.019
THBS1	0.051 ± 0.039	0.058 ± 0.049	0.836 ± 1.118	<0.001	0.259	<0.001	<0.001
UCHL1	0.041 ± 0.023	0.044 ± 0.010	0.549 ± 0.614	<0.001	0.08	<0.001	0.016
DLEC1	0.189 ± 0.065	0.203 ± 0.073	0.989 ± 0.767	0.012	0.662	0.005	0.033

SD, standard deviation; GC, gastric cancer; CAG, chronic atrophic gastritis; ^a^ *p* value calculated by nonparametric Kruskal-Wallis test; ^b^ *p* value calculated by Bonferroni correction.

**Table 3:** Receiver-operating characteristic analysis of methylated genes for diagnosis of GC.

Gene	Number of controls/CAG samples without DNA methylation detected/total number of controls/CAG samples (%)	Number of GC samples with DNA methylation detected/total number of GC samples (%)	Specificity	Sensitivity	AUC (SE)	95% CI
FLNC	80/86 (93.0)	55/82 (67.1)	93.0	67.1	0.718 (0.037)	0.65–0.79
THBS1	81/86 (94.2)	52/82 (63.4)	94.2	63.4	0.706 (0.038)	0.63–0.78
UCHL1	77/86 (89.5)	46/82 (56.1)	89.5	56.1	0.663 (0.041)	0.60–0.73
DLEC1	80/86 (93.0)	66/82 (80.5)	93.0	80.5	0.868 (0.033)	0.80–0.91

GC, gastric cancer; CAG, chronic atrophic gastritis; AUC, area under the curve; SE, standard error; CI, confidence internal.

**Table 4:** Correlations between clinicopathological features and methylation status of genes in GC patients.

Variables	GC patients	FLNC		THBS1		UCHL1		DLEC1	
	*n* = 82	M (%)	*p* ^a^	M (%)	*p* ^a^	M (%)	*p* ^a^	M (%)	*p* ^a^
Age (years)			0.953		**0.021**		0.429		0.396
<60	28 (34)	18 (64)		13 (46)		14 (50)		24 (86)	
≥60	54 (66)	37 (69)		39 (72)		32 (60)		42 (78)	
*H. pylori* infection			**0.009**		0.106		0.065		0.747
No	38 (46)	20 (53)		22 (58)		20 (53)		30 (79)	
Yes	44 (54)	35 (80)		30 (68)		26 (59)		36 (81)	
Tumor size (cm)			0.185		0.797		0.974		**0.040**
<3	34 (42)	20 (59)		21 (62)		19 (56)		28 (82)	
≥3	48 (58)	35 (73)		31 (65)		27 (56)		38 (79)	
Differentiation			**0.016**		0.223		0.305		0.766
Good/moderate	54 (66)	35 (65)		35 (65)		31 (57)		47 (87)	
Poor	28 (34)	20 (71)		17 (61)		15 (54)		19 (68)	
TNM Stage			0.131		0.262		**0.026**		0.361
I, II	48 (59)	29 (60)		28 (58)		22 (46)		37 (77)	
III, IV	34 (41)	26 (76)		24 (71)		24 (71)		29 (85)	
Lymph node metastasis			0.233		0.854		**0.014**		0.705
N0	29 (35)	17 (59)		18 (62)		11 (38)		24 (83)	
N1–3	53 (65)	38 (72)		34 (64)		35 (66)		42 (79)	

M, methylation, *n* (%); ^a^ *p* value calculated by Fisher's exact test; *p* values < 0.05 are presented in bold.

**Table 5:** Clinical characteristics of patients correlate with overall survival by Cox proportional hazard regression analysis.

Variables	Univariate analysis			Multivariate analysis		
	HR	95% CI	*p* value	HR	95% CI	*p* value
Age (<60 y, ≥60 y)	3.821	1.104–13.224	**0.034**	0.990	0.222–4.412	0.990
Gender (female, male)	0.966	0.375–2.493	0.943			
Alcohol consumption (no, yes)	1.774	0.665–4.732	0.252			
*H. pylori* infection (no, yes)	2.076	0.740–5.826	0.165			
Histological type (intestinal, diffuse)	0.435	0.126–1.506	0.189			
Differential (good/moderate, poor)	3.422	1.321–8.861	**0.011 **	2.223	0.865–6.232	0.095
TNM stage (I + II, III + IV)	16.434	3.760–51.834	**0.000 **	10.799	2.013–37.938	**0.005**
Lymph node metastasis (N0, N1–3)	1.988	0.653–6.054	0.226			
Tumor size (<3 cm, ≥3 cm)	1.198	0.450–3.194	0.717			
FLNC methylation (no, yes)	3.798	0.416–26.502	0.132			
THBS1 methylation (no, yes)	3.262	0.943–11.285	0.062			
UCHL1 methylation (no, yes)	6.694	1.536–19.172	**0.011**	3.688	0.769–14.677	0.103
DLEC1 methylation (no, yes)	4.162	0.553–31.308	0.166			

HR, hazard ratio; CI, confidence interval; *p* values < 0.05 are presented in bold.

## References

[1] Shi Y., Zhou Y. E. (2010). The role of surgery in the treatment of gastric cancer. *Journal of Surgical Oncology*.

[2] Kamangar F., Dores G. M., Anderson W. F. (2006). Patterns of cancer incidence, mortality, and prevalence across five continents: defining priorities to reduce cancer disparities in different geographic regions of the world. *Journal of Clinical Oncology*.

[3] Correa P. (1988). A human model of gastric carcinogenesis. *Cancer Research*.

[4] Dong C.-X., Deng D.-J., Pan K.-F. (2009). Promoter methylation of p16 associated with helicobacter pylori infection in precancerous gastric lesions: a population-based study. *International Journal of Cancer*.

[5] McCabe D. C., Caudill M. A. (2005). DNA methylation, genomic silencing, and links to nutrition and cancer. *Nutrition Reviews*.

[6] Zou X.-P., Zhang B., Zhang X.-Q., Chen M., Cao J., Liu W.-J. (2009). Promoter hypermethylation of multiple genes in early gastric adenocarcinoma and precancerous lesions. *Human Pathology*.

[7] Lee T.-L., Leung W. K., Chan M. W. Y. (2002). Detection of gene promoter hypermethylation in the tumor and serum of patients with gastric carcinoma. *Clinical Cancer Research*.

[8] Leung W. K., To K.-F., Chu E. S. H. (2005). Potential diagnostic and prognostic values of detecting promoter hypermethylation in the serum of patients with gastric cancer. *British Journal of Cancer*.

[9] Kaneda A., Kaminishi M., Yanagihara K., Sugimura T., Ushijima T. (2002). Identification of silencing of nine genes in human gastric cancers. *Cancer Research*.

[10] Yamashita K., Park H. L., Kim M. S. (2006). PGP9.5 methylation in diffuse-type gastric cancer. *Cancer Research*.

[11] Kang G. H., Shim Y.-H., Jung H.-Y., Kim W. H., Ro J. Y., Rhyu M.-G. (2001). CpG island methylation in premalignant stages of gastric carcinoma. *Cancer Research*.

[12] Zhang Y., Ye X., Geng J., Chen L. (2010). Epigenetic inactivation of deleted in lung and esophageal cancer 1 gene by promoter methylation in gastric and colorectal adenocarcinoma. *Hepato-Gastroenterology*.

[13] Dalkilic I., Schienda J., Thompson T. G., Kunkel L. M. (2006). Loss of filaminC (FLNc) results in severe defects in myogenesis and myotube structure. *Molecular and Cellular Biology*.

[14] Ying J., Poon F. F., Yu J. (2009). *DLEC*1 is a functional 3p22.3 tumour suppressor silenced by promoter CpG methylation in colon and gastric cancers. *British Journal of Cancer*.

[15] Kang G. H., Lee S., Kim J.-S., Jung H.-Y. (2003). Profile of aberrant CpG island methylation along multistep gastric carcinogenesis. *Laboratory Investigation*.

[16] Lee Y. M., Lee J. Y., Kim M. J. (2006). Hypomethylation of the protein gene product 9.5 promoter region in gallbladder cancer and its relationship with clinicopathological features. *Cancer Science*.

[17] Washington K. (2010). 7th edition of the AJCC cancer staging manual: stomach. *Annals of Surgical Oncology*.

[18] Eads C. A., Danenberg K. D., Kawakami K. (2000). MethyLight: a high-throughput assay to measure DNA methylation. *Nucleic Acids Research*.

[19] Tan S.-H., Ida H., Lau Q.-C. (2007). Detection of promoter hypermethylation in serum samples of cancer patients by methylation-specific polymerase chain reaction for tumour suppressor genes including RUNX3. *Oncology Reports*.

[20] Abbaszadegan M. R., Moaven O., Sima H. R. (2008). p16 promoter hypermethylation: a useful serum marker for early detection of gastric cancer. *World Journal of Gastroenterology*.

[21] Stossel T. P., Condeelis J., Cooley L. (2001). Filamins as integrators of cell mechanics and signalling. *Nature Reviews Molecular Cell Biology*.

[22] Wilkinson K. D., Lee K. M., Deshpande S., Duerksen-Hughes P., Boss J. M., Pohl J. (1989). The neuron-specific protein PGP 9.5 is a ubiquitin carboxyl-terminal hydrolase. *Science*.

[23] Okochi-Takada E., Nakazawa K., Wakabayashi M. (2006). Silencing of the *UCHL1* gene in human colorectal and ovarian cancers. *International Journal of Cancer*.

[24] Tian F., Yip S. P., Kwong D. L. W., Lin Z., Yang Z., Wu V. W. C. (2013). Promoter hypermethylation of tumor suppressor genes in serum as potential biomarker for the diagnosis of nasopharyngeal carcinoma. *Cancer Epidemiology*.

[25] Tokumaru Y., Yamashita K., Myoung S. K. (2008). The role of PGP9.5 as a tumor suppressor gene in human cancer. *International Journal of Cancer*.

[26] Zhang Y., Miao Y., Yi J., Wang R., Chen L. (2010). Frequent epigenetic inactivation of deleted in lung and esophageal cancer 1 gene by promoter methylation in non-small-cell lung cancer. *Clinical Lung Cancer*.

[27] Qin Q., Qian J., Ge L. (2014). Effect and mechanism of thrombospondin-1 on the angiogenesis potential in human endothelial progenitor cells: an in vitro study. *PLoS ONE*.

[28] Kim H. C., Roh S. A., Ga I. H., Kim J.-S., Yu C. S., Kim J. C. (2005). CpG island methylation as an early event during adenoma progression in carcinogenesis of sporadic colorectal cancer. *Journal of Gastroenterology and Hepatology*.

[29] Liu B.-L., Cheng J.-X., Zhang W. (2010). Quantitative detection of multiple gene promoter hypermethylation in tumor tissue, serum, and cerebrospinal fluid predicts prognosis of malignant gliomas. *Neuro-Oncology*.

[30] Mizukami H., Goto T., Kitamura Y. (2009). PGP9.5 was less frequently methylated in advanced gastric carcinoma. *Hepato-Gastroenterology*.

[31] Mizukami H., Shirahata A., Goto T. (2008). PGP9.5 methylation as a marker for metastatic colorectal cancer. *Anticancer Research*.

[32] Mandelker D. L., Yamashita K., Tokumaru Y. (2005). PGP9.5 promoter methylation is an independent prognostic factor for esophageal squamous cell carcinoma. *Cancer Research*.

[33] Trifa F., Karray-Chouayekh S., Jmaa Z. B. (2013). Frequent CpG methylation of ubiquitin carboxyl-terminal hydrolase 1 (UCHL1) in sporadic and hereditary Tunisian breast cancer patients: clinical significance. *Medical Oncology*.

[34] Kagara I., Enokida H., Kawakami K. (2008). CpG hypermethylation of the UCHL1 gene promoter is associated with pathogenesis and poor prognosis in renal cell carcinoma. *Journal of Urology*.

[35] Guerrero D., Guarch R., Ojer A. (2008). Hypermethylation of the thrombospondin-1 gene is associated with poor prognosis in penile squamous cell carcinoma. *BJU International*.

[36] Kang G. H., Lee H. J., Hwang K. S., Lee S., Kim J.-H., Kim J.-S. (2003). Aberrant CpG island hypermethylation of chronic gastritis, in relation to aging, gender, intestinal metaplasia, and chronic inflammation. *American Journal of Pathology*.

[37] Chan W.-H., Chang K.-P., Yang S.-W. (2010). Transcriptional repression of DLEC1 associates with the depth of tumor invasion in oral squamous cell carcinoma. *Oral Oncology*.

[38] Wang Z., Yuan X., Jiao N., Zhu H., Zhang Y., Tong J. (2012). CDH13 and FLBN3 gene methylation are associated with poor prognosis in colorectal cancer. *Pathology and Oncology Research*.

[39] Sasaki H., Hikosaka Y., Kawan O., Moiyama S., Yan M., Fujii Y. (2010). Methylation of the DLEC1 gene correlates with poor prognosis in Japanese lung cancer patients. *Oncology Letters*.

[40] Maekita T., Nakazawa K., Mihara M. (2006). High levels of aberrant DNA methylation in *Helicobacter pylori*-infected gastric mucosae and its possible association with gastric cancer risk. *Clinical Cancer Research*.

